# Siloed mentality, health system suboptimization and the healthcare symphony: a Canadian perspective

**DOI:** 10.1186/s12961-024-01168-w

**Published:** 2024-07-17

**Authors:** Robin S. Lau, Mari E. Boesen, Lawrence Richer, Michael D. Hill

**Affiliations:** 1 Alberta Innovates, #1500, 10104 103 Ave NW, Edmonton, AB T5J 0H8 Canada; 2 Calgary Centre for Clinical Research (CCCR), University of Calgary, 400, Cal Wenzel Precision Health, 3280 Hospital Drive NW, Calgary, AB T2N 4Z6 Canada; 3 Faculty of Medicine and Dentistry, College of Health Sciences, Edmonton Clinic Health Academy, 3-372, 11405-87 Ave, Edmonton, AB T6G 1C9 Canada; 4 Calgary Stroke Program, Department of Clinical Neuroscience and Hotchkiss Brain Institute, Health Sciences Centre, University of Calgary, RM 2939, 3330 Hospital Drive NW, Calgary, AB T2N 4N1 Canada

**Keywords:** Health services and policy research, Healthcare, Learning health systems, Quality improvement, Organizational culture, Situated learning, Complex adaptive systems, Continuous improvement, Patient outcomes

## Abstract

Measuring and optimizing a health system is challenging when patient care is split between many independent organizations. For example, patients receive care from their primary care provider, outpatient specialist clinics, hospitals, private providers and, in some instances, family members. These silos are maintained through different funding sources (or lack of funding) which incentivize siloed service delivery. A shift towards prioritizing patient outcomes and keeping the patient at the centre of care is emerging. However, competing philosophies on patient needs, how health is defined and how health is produced and funded is creating and engraining silos in the delivery of health services. Healthcare and health outcomes are produced through a series of activities conducted by diverse teams of health professionals working in concert. Health professionals are continually learning from each patient interaction; however, silos are barriers to information exchange, collaborative evidence generation and health system improvement. This paper presents a systems view of healthcare and provides a systems lens to approach current challenges in health systems. The first part of the paper provides a background on the current state and challenges to healthcare in Canada. The second part presents potential reasons for continued health system underperformance. The paper concludes with a system perspective for addressing these challenges.

## Introduction

The global pandemic caused by severe acute respiratory syndrome coronavirus 2 (SARS-CoV-2) pushed health systems past their capacity limit globally, and exposed existing system inefficiencies. Canadian hospitals have been operating at 100–120% capacity and this continued overloading undermines their ability to deliver safe, effective and timely care [1]. Vaccines lower the risk of getting and spreading SARS-CoV-2 and prevent serious illness and death, yet vaccine hesitancy both during and continuing postpandemic have limited their public health benefit. Delayed or cancelled surgeries were common during the pandemic. Temporary hospital units were created to ease overcrowding and overcapacity, and healthcare workers retired from the workforce, quit or moved to alternate employment, often due to burnout, stress, anxiety and depression in dedicated frontline workers [1]. This led to shortages which exacerbated capacity issues and in a vicious cycle created heavy workloads resulting in more burnout [1]. Four years after the WHO declared a pandemic (11 March 2020), health systems are still struggling. The global pandemic exaggerated existing problematic structures and put health systems into the spotlight. Our health systems have a complex role in promoting, restoring, and maintaining health of people and populations but they are falling short as they face increasing patient demand and pressures to perform.

Performance and cost are related. Health expenditures as a share of gross domestic product (GDP) are trending upwards in Canada [2]. Among the 36 countries that make up the Organization for Economic Cooperation and Development (OECD), in 2023, Canada spent an estimated 12.1% of GDP, or $8740 CAD per capita on healthcare and is among the highest spenders, above the OECD average of 9.7% of GDP or $6044 CAD per capita in 2021 [3, 4]. In return, Canada’s healthcare performance ranks second to last; the 2021 Commonwealth Fund report ranked Canada tenth of eleven countries performing poorly in access, equity and healthcare outcome categories – only ahead of the United States [5]. Technological and pharmacological advances, increased demand and public expectations, prescription drug expenditures and an ageing population are drivers of increased healthcare spending [6].

Health systems are complex adaptive systems, where health system performance and behaviours change over time [7]. Healthcare settings, people and processes evolve dynamically in terms of relationships and interactions [8]. The nonlinearity of complex adaptive systems results in numerous ways to produce the same health outcomes. Empiric data and evidence are required – the clues to health improvement and population health must be observed from the differences within and between populations over time [9].

In this early postpandemic vantage point, we explore the Canadian health system as an exemplar to try to understand the path to better performance and better outcomes at both the individual and the societal level. Our underlying thesis is that health systems are complex adaptive systems whose components are not necessarily additive.

### The problem

The Canadian health system is still not meeting the needs of the populations they serve. It is experiencing physician, nurse and healthcare worker shortages [10]; increasing wait times [11]; reduced preventive care service delivery [12] and increasing costs [2]. A fundamental problem is suboptimization, where productivity improvements in one area (silo) can result in system level decreases in productivity [13]. A common assumption is that a higher unit level productivity will result in a higher system level productivity [13] or that maximizing patient outcomes through disease-specific clinical practice guidelines result in superior patient well-being and outcomes. However, real life observations on patient outcomes [14–16] and health system performance outcomes [3–5] indicate otherwise. The disconnect between individual patients, practitioners, organizations and system outcomes are due to an unclear link between production and value [13].

This discordance arises, in part perhaps due to the priorities which lead to measurement at different levels of the health system. Health system stakeholders have numerous, sometimes conflicting goals around access to services, cost reduction, quality, cost containment, safety, patient centredness and satisfaction [17]. Healthcare units and stakeholders usually define performance outcomes based on discrete patient needs and demands. For example, the health unit level may use ratio-based measures such as labour-hours per patient or care episode and the number of resources consumed. At the organizational level, performance metrics measure hospital productivity, wait times, unit capacity or number of procedures performed. At the system or macro level, performance metrics can be health expenditures as a share of gross domestic product or broadly defined measures such as access, safety and quality of care. Siloed outcomes between units and organizations cannot be efficient from a systems perspective [13]. A holistic integrated systems approach to patient care and productivity can align health system stakeholders to provide the best health services from a system perspective [13, 17].

## Health systems

Health systems that were historically devoted to prevention and treatment of infectious diseases and discrete episodes of acute care are now faced with the management of chronic health conditions such as heart disease, diabetes mellitus, chronic obstructive pulmonary disease and many types of cancer [18]. In the early eighteenth-century health systems focus on the isolation of the ill and quarantining of the exposed to contain contagious diseases [19]. These systems evolved into voluntary general hospitals for the physically ill and institutions for the mentally ill in the late eighteenth century [19]. The nineteenth century brought about the importance of sanitation as a mechanism to address the causes of disease and the vehicle for transmission [19]. Diseases were considered an “indicator of a societal problems as well as a personal problem” [19].

Improvements in technology, medical care and public health policy have resulted in increased survival rates from previously fatal health conditions. Declining infectious disease deaths resulting in longer life expectancies and the demographic trends of larger numbers of births post-second world war and now declining fertility rates globally have resulted in an increasing proportion of older citizens and a greater segment of the population with multiple age-associated concurrent medical conditions (multimorbidity) [20, 21]. These chronic conditions are not so much curable as manageable. This makes multimorbidity both costly and associated with increased health system utilization and poorer health outcomes [22–25]. Individuals with multimorbidity have increased risks of receiving less than the best practice care [26, 27], higher healthcare costs, increased polypharmacy, and longer and more frequent hospitalizations [28]. Significant inefficiencies emerge in health systems, as systems built to cope with single acute illnesses try to cope with new and more complex demands. Excess costs are caused by overuse of unnecessary health services or technologies, inefficiently delivered health services, medical errors, mispriced health technologies and missed prevention opportunities [29].

Reductionist approaches to each multimorbid condition can leave patients to navigate a complex health system and encounter fragmentation of care, duplication of services and treatment errors [30]. Coordinating and communicating needs to different providers can be overwhelming, resulting in poor health outcomes [30]. This fragmented, disease-centric care is maintained through clinical practice guidelines oriented towards single diseases and physician incentives, including fee-for-service healthcare professional remuneration linked to discrete International Classification of Disease (ICD) codes [31]. The result is that the specialist is responsible for and focuses upon a single disease among a patient’s many chronic conditions and not the whole patient [31]. Basing care on disease-specific clinical practice guidelines poses care challenges for patients with multimorbidity and may have unintended effects [32].

The complexity of the interaction between the patient and health system means that healthcare is a complex adaptive system comprising a network of components (stakeholders, organizations, and so on) interacting nonlinearly at different levels (patient, provider, organizational, policy) producing system performance and behaviours that cannot be completely understood by knowing about individual components [7, 33]. Complex adaptive systems can produce the same outcomes in numerous different ways or numerous different outcomes in the same ways. What works in one healthcare setting may not work in another; healthcare improvement is context dependent [34].

## Complex adaptive systems (CAS)

Complex adaptive systems (CAS) do not operate in the same way as mechanical systems [35]. Mechanical systems (1) can be decomposed and recomposed and (2) are overseen by a single entity with the authority and resources to design the system [35]. Components of mechanical systems interact linearly to produce predictable outcomes or outputs, which can be controlled by manipulating each component of the system [33]. Whereas CAS are comprised of networks of components (healthcare organizations, healthcare decision-makers, healthcare professionals, healthcare providers, patients, the general public) that interact nonlinearly and produce unexpected results [33]. Stakeholders with diverse interests are layered by organization, specialty and location [35]. The health system is a social system, the most complex class of systems [36], composed of a “patterned series of interrelationships existing between individuals, groups, and institutions forming a coherent whole” [37]. Regulations and policy trying to control behaviour often result in unintended consequences [33].

Behaviours of independent health system agents are motivated by physical, psychological or social rules rather than system demands and needs – resulting in goals and behaviours that are likely in conflict [35]. As each agent learns over time, they modify and adapt their behaviours, leading to self-organizing units – behavioural patterns emerge rather than being designed into the system [35]. The nonlinearity of CAS results in numerous ways to produce health outcomes. Given the complexity, decomposition of complex adaptive systems may result in the loss of important contextual information critical to understanding the phenomenon under study [35].

With increasing pressures to reduce health system inefficiencies and improve health outcomes, there is an urgency to understand where the system failures are, what causes them, what maintains system inertia (that is, behaviours, processes and so on), and what can be done to achieve health system goals. By viewing healthcare from a systems perspective and a CAS, several intrinsic system properties can be used: (1) to understand how certain decisions affect the dynamic behaviour of the system and (2) to inform implementation and decision-making [33].

Box 1: defining characteristics of complex adaptive systems Complex adaptive systems have the following characteristics (adapted from Rouse [35]):

- “Nonlinear and dynamic and do not inherently reach fixed equilibrium points. As a result, system behaviours may appear to be random or chaotic”.
- “Composed of independent agents whose behaviour is based on physical, psychological or social rules rather than the demands of system dynamics”.
- “Because agents’ needs or desires, reflected in their rules, are not homogenous, their goals and behaviours are likely to conflict. In response to these conflicts or competitions, agents tend to adapt to each other’s behaviours”.
- “Agents are intelligent. As they experiment and gain experience, agents learn and change their behaviours accordingly. Thus, overall system behaviour inherently changes over time”.
- “Adaptation and learning tend to result in self-organization. Behaviour patterns emerge rather than being designed into the system. The nature of emergent behaviours may range from valuable innovations to unfortunate accidents”.
- “There is no single point of control. System behaviours are often unpredictable and uncontrollable, and no one is “in charge”. Consequently, the behaviours of complex adaptive systems can usually be more easily influence than controlled”.

## Resilience engineering

High-functioning health systems will be resilient to external shocks. The health system handling of the pandemic and poor health system indicators [1, 2, 38, 39] may indicate a failure of health systems to handle disruptions and variations to normal operations. Adaptability and flexibility of human work and the ability to adjust to local conditions, shortcomings or quirks of technology and to predictable changes in resources and demands allows for the system to function efficiently [40]. The performance variability is necessary to cope with the complexity of the real world [40]. Failures occur when system adjustments to harmful influences are insufficient or when the demands push the system beyond its performance variability [40]. This concept of failure is not due to a single traceable cause or factor, but to an inability of the system to cope and adapt quickly enough to system demands.

Resilience engineering is “the deliberate design and construction of systems that have the capacity of resilience. Resilient systems typically experience disturbances” [41]. Resilience is “how well can a system handle disruptions and variations that fall outside of the base mechanisms/model for being adaptive as defined in that system” [42]. There are four aspects to resilience: (1) monitoring, scanning, listening, observing, attending to, exploring and examining system operation to understand current state; (2) responding, reacting, intervening, correcting, tuning, adjusting, tweaking and trading off goals in response to events or conditions; (3) anticipating, projecting, predicting and foreseeing future events and conditions; and (4) learning, incorporating, reviewing, integrating and reorganizing system knowledge [41, 43]. Managing disturbances to system operations – resilience – requires trade-offs across various goals [41].

Meeting patient needs and increasing patient safety requires greater focus on the unit of healthcare production, human experts and an increasing focus on the interdependencies between various care providers and social supports for high quality coordinated care. Training and development of human resources and professional development improves the skills needed to manage disturbances and incorporates a culture of continual learning [41]. In addition, an updated systems model and understanding of the complexity of care and how care is provided is needed– from simple linear thinking to a systemic model [40].

Resilience in organizations and/or systems in healthcare is a human phenomenon, which relies on human experts for direction and control and coping with complexity and uncertainty [41]. Crisis and uncertainty often push health systems to revert to the protection of core competencies. It is difficult to change, adapt and be resilient when healthcare providers are facing pressures on all fronts with organizational demands of efficiency (to do more with less), increasing needs to keep up with professional development and a never-ending supply of complex patients. Designing resilience requires attention to the healthcare professionals training and development, well-being and increasing system role. This requires strategy and combining activities for strategic fit [44].

Fit is how activities reinforce one another [44]. Porter [44] defines three types of fit: (1) simple consistency (first order), (2) when activities are reinforcing (second order) and (3) optimization of effort (third order). First-order fit is consistency and aligns all healthcare activities with the purpose of healthcare and health systems which is “to promote, restore or maintain health” [44, 45]. Inconsistent and conflicting goals at different levels of the health system erode consistency of purpose. Second-order fit is where activities reinforce each other [44] and is where all activities in the care of patients with the same condition are coordinated from hospital into the community. Third-order fit is optimizing efforts through (1) the coordination and information exchange across activities and (2) design choices of healthcare activities across silos (healthcare organizations, not-for-profits, communities, patients and so on) to eliminate redundancy and wasted effort [44]. Fit is about systems, where resilience is a product of the system and cannot be attributed to an individual part.

## The social determinants of health and health inequities

Health systems exist in a social context. Ethno-cultural factors and socioeconomic status play a critical role in health. Health improvement happens in the context of the social determinants of health, beyond the health system. These include “the conditions in which people are born, grow, work, live and age, and the wider set of forces and systems shaping the conditions of daily life” [46]. The social determinants of health refer to social, economic, environmental and demographic factors that influence health outcomes. In current thinking, there is a large emphasis on the proximate risk factors or risk factors that are controllable at the individual level and social factors whose influences on a specific disease have received far less attention [47]. The focus on proximate causes of disease – such as diet, hypertension, cholesterol, lack of exercise – are due to the controllable, manipulative nature of such factors. This simplistic view assumes that if the risk factors, the individual risk behaviours are eliminated as causal agents, mortality and illness should be reduced [48]. However, less-modifiable social determinants of health may contribute a larger attributable risk fraction to disease and illness.

Structuring the health system to only address individual risk factors may improve the current situation in the short term, however, will not translate into improvement in health outcomes or lasting reductions in mortality and illness. Researchers, decision- and policy-makers need to think about individual risk factors in the context of the social determinants of health [47]. The promotion of the individual as the root of the problem in health policy avoids the much needed debate about how income distribution, the environment and the medical establishment influence disease and health status [49]. There are numerous social, cultural and economic factors driving and shaping the behaviours and habits of individuals.

Investigating socioeconomic differences among populations and their associations to healthcare utilization may provide insight into the fundamental determinants of health and contribute to initiatives aimed at improving population health [50]. For example, what is the relationship between social determinants and the effectiveness of health interventions aimed at improving access, health outcomes and population health? Are health interventions which are intended to improve health outcomes modified or made less effective because of some aspects of income or education? A health system underpinned by robust data systems and networks creates the potential to study the larger health system and aid in the refining of health system priorities for healthcare improvements. It would allow for the implementation of prevention strategies in high-risk conditions/patients but also a population health approach [51].

A population health strategy [51] shifting the population distribution of risk factors would require collaboration and partnership with various organizations and communities to address high-risk individual health factors (that is, obesity, high blood pressure, inactivity and so on) and the social determinants of health. Healthcare is a team sport; health systems have reached the limit on what each healthcare profession can contribute individually. Overcoming organizational and health system inertia is needed.

## Silos: barriers to system change

Health systems face a complex coordination problem because of the historical and reductionist focus on single illnesses. How can health systems be designed to meet patients’ evolving needs which include managing their chronic conditions for improved quality of life? Methods and structures that appear practical for dividing care from one perspective result in silos that become barriers to other aspects of care delivery. These silos of care are a major barrier to innovation and health system change, made especially evident throughout the pandemic. Extreme examples are economic units of healthcare delivery. Acute stroke patients have benefitted substantially by investment in hyperacute care. Costs are higher at the front end but downstream savings are dramatically higher, so overall care is cheaper. Acute stroke treatment is highly cost effective. However, budgetary silos prevent downstream savings from being applied to higher acute care costs.

Silos are a metaphor used to illustrate pockets of interaction and knowledge in organizations [52]. These represent barriers to communication and information exchange, often cited as obstructions to collaboration and coordination. Organizational silos are vast psychological spaces of compartmentalization, segregation and differentiation [52]. In healthcare, organizational silos can be disciplinary or professional, based on where people research, work, collaborate and function daily. For example, silos can include medical doctors, nurses, researchers, healthcare professionals and healthcare leaders, and their specific discipline/specialization or organizational department, each working in their own institutional space.

These silos influence the perspectives of an organization’s units and its team members. Silos exist in the minds of the employees who have a shared understanding of their organization, unit or team’s goals and objectives [52]. Siloed mentality is characterized by (1) not knowing what others are doing, (2) stuckness, (3) isolation, (4) powerlessness and (5) lack of trust, respect, collegiality and collaboration [52]. Siloed mentality influences work behaviour and creates an “us versus them” mindset and impacts how departments and divisions treat each other [52]. This behaviour is often unconscious and members are unaware of this type of reaction to other units, teams or organizations [52]. Silo mentality “results in the creation of barriers to communication and in the development of disjointed work processes with negative consequences to the organization, employees and patients” [53].

Silos often focus on their own disciplinary space and attempt to optimize business processes within their organizational unit. However, focusing on discrete components of the health system and ignoring the larger consequences leads to system suboptimization, which results in the long-term erosion of health outcomes at the expense of the patient. Where individual action without connection to the system decreases benefits for all, patients bear the consequences through poor care coordination and poor health outcomes.

As populations age, the challenge to health systems is in the health services coordination for chronic health conditions management for better patient quality of life. The primary goal is improving the continuity of care for patients, from acute to the community. This requires (1) increased collaboration and coordination of diverse care providers and organizations along the patient’s care journey, (2) knowledge generation across disciplinary boundaries and (3) increased data flow for health system learning and feedback. Silos are a barrier to collaboration, cross-disciplinary knowledge generation and health system learning.

## Health and the healthcare symphony

Recommendations for health system knowledge generation across silos cite a culture shift towards continuous learning and quality improvement [54]. Schein [55] states that culture “is a pattern of shared basic assumptions learned by a group as it solved its problems of external adaptation and internal integration, which has worked well enough to be considered valid and therefore to be taught to new members as the correct way to perceive, think, and feel in relation to those problems”. The engrained cultural patterns designed around short-term needs and acute demands are having trouble coping with the shift to an increasing prevalence of chronic diseases and mental illnesses requiring long-term management and planning. Long-term management of complex patients requires a diverse team of healthcare professionals and involves coordinating care across disease silos. The health system’s inability to cope has eroded health system performance [3–5, 39].

Collaborative healthcare action produces better health outcomes. A single healthcare professional cannot produce health outcomes alone; only a carefully coordinated and sequenced set of actions by a team of several health professionals working in concert can produce patient health outcomes. The production of health outcomes can only be meaningfully attributed to a healthcare team or health system. In health systems, individual knowledge does not explain health system activities where (1) teams are required to achieve goals (individual knowledge is necessary but not sufficient) and (2) sustained organizational activities where many different individuals are involved and do not necessarily act together [56]. This suggests that knowledge is social and “shaped by the context(s) in which it is acquired and used” [56], supporting the concept of situated learning. Learning is “always situated in a particular context which comprises not only a location and a set of activities in which knowledge either contributes or is embedded, but also a set of social relations which give rise to those activities” [56].

What is needed is a shared knowledge space for individuals to interact, reflect and learn [57]. The greater the shared knowledge space, the greater the understanding of each other’s roles and responsibilities, and the less context is needed to share knowledge [57]. Small existing shared knowledge space (for example, silos) require a greater need for contextual information and more effort is needed to exchange information [57]. One way to recreate context and provide a shared knowledge space for evidence generation, learning and health system improvement is through small, manageable health improvement projects (for example, pragmatic clinical trials, quality improvement initiatives) spanning disciplines and organizations.

## The pursuit of quality

The underperformance of the health system may be caused by focusing on suboptimal systems and by focusing on short term efficiency goals (for example, cost reduction, control and containment) at the expense of long-term adaptability; also known as the productivity dilemma [58, 59]. The fundamental objectives of health systems is to improve the health of populations, improve the responsiveness of the health system to the population it serves and promote fairness in financial contribution [60]. Therefore, the overt emphasis on efficiency has led to a focus on time and cost management and using technology to increase efficiency – to do more with fewer resources. However, this focus on efficiency distracts from the concept of quality and long-term adaptability.

In healthcare, quality is determined by what the customer or stakeholder needs from a specific service, product, or project. Quality in healthcare is meeting the needs of patients that utilize the health system. Health systems are designed for improving patients’ health and well-being; quality is keeping the patient at the centre of care with activities contributing to improving their well-being. A high-quality health system is responsive and meets patient needs. Combining patient quality outcomes with productivity metrics from the unit, organization and system can help overcome system suboptimization [13]. There needs to be mechanisms to gather patient requirements into how the health system can meet their needs better and more effectively given the constraints to patients, families and caregivers, healthcare providers, healthcare organizations (including not-for-profit) and health systems.

## What does success look like?

A successful health system may be likened to an orchestra playing a complex symphony. The orchestra is composed of a diverse ensemble of interdependent instrument sections much like the health system depends on numerous operational sections from janitorial services, health professionals, to leadership. Unlike an orchestra, the health system also depends on external players such as not-for-profit organizations, community organizations and family/caregivers to realize optimal health outcomes. Coordinating work among sections within the system and with external providers is exceptionally complex requiring an understanding of each section’s roles and responsibilities and overall contributions to the “orchestra”.

Suboptimization or optimizing the outcome for a single subsystem (or silo) (for example, the trumpet section) does not optimize the outcomes for the system as a whole. Maintaining silos, the fear of change and lack of adaptation to new health system challenges, can stem from the fear of a loss of identity. Each part of an orchestra has an integral role to play: health systems and healthcare providers each have an integral role to produce high quality health outcomes; ones role cannot exist without the others. The new challenge becomes, how do groups/teams/organizations/units maintain identity in the face of new health system challenges? To maintain identity and to evolve as a specialty, health systems need to learn and set goals collectively (that is, which symphony will be performed?). Creating a culture of continual learning is a way to evolve the current identity and practice addressing the new health system challenges of today and in the future.

Short-term success will be to identify, prioritize and create a shared vision of what a successful health system could look like and what parameters or outcomes would define success. The recognition of the reality of single disease, guideline-based care that results in silos of care is essential to develop that success. The goal is transforming high-priority clinical process into a resilient learning health system: (a) pick a high priority clinical process, (b) build an evidence based best practice guideline around that process, (c) blend the guideline into a clinical workflow parallel with a data system that is going to track what happens and (d) feed the data into a ‘lean learning loop’ [61]. The learning health system has been recommended as a rallying point to promote the learning health culture necessary to high performing health systems.

## Data Availability

Not applicable.

## Abbreviations

AbSPORU: Alberta Strategy for Patient Oriented Research Support Unit
ICD: International classification of disease
QI: Quality improvement
SARS-CoV-2: Severe acute respiratory syndrome coronavirus 2
